# Vertical Dimension in Prosthodontics Theory and Practice (Part III): Contemporary Clinical Protocols and Decision-Making for Loss of Vertical Dimension of Occlusion

**DOI:** 10.7759/cureus.109416

**Published:** 2026-05-22

**Authors:** Mostafa I Fayad, Rania Moussa, Esmail A Abdel-Gawwad, Ahmed A Laithy, Ehab Esmail Mahmoud, Ahmed Atef Shon, Suad M Hassan, AbdElrahman Karam Eldain, Belal Alwakeel, Mohamed A Helal

**Affiliations:** 1 Substitutive Dental Sciences Department, College of Dentistry, Taibah University, Madinah, SAU; 2 Removable Prosthodontics Department, Faculty of Dental Medicine, Al-Azhar University, Cairo, EGY; 3 Restorative Dental Sciences Department, College of Dentistry, Taibah University, Madinah, SAU; 4 Dentistry Department, Mouwasat Hospital, Riyadh, SAU; 5 Dentistry Department, Al Mouwasat Hospital, Madinah, SAU

**Keywords:** clinical decision-making, dahl concept, full-mouth rehabilitation, occlusal rehabilitation, vertical dimension of occlusion

## Abstract

Loss of vertical dimension of occlusion (VDO) is among the most clinically significant presentations in prosthodontics and restorative dentistry. Despite its profound impact on orofacial function, esthetics, masticatory efficiency, and oral health-related quality of life, no universally accepted operational definition, diagnostic threshold, or treatment protocol currently exists. This narrative literature review aimed to review the contemporary evidence on the clinical protocols and decision-making in the rehabilitation of patients with VDO loss. This comprehensive narrative review was conducted in accordance with the Scale for the Assessment of Narrative Review Articles (SANRA) reporting principles. PubMed/MEDLINE, the Cochrane Library, Scopus, and Web of Science were searched from inception to October 2025 using combinations of Medical Subject Headings (MeSH) and free-text terms including “vertical dimension of occlusion”, “tooth wear”, “erosive tooth wear”, “bruxism”, “occlusal rehabilitation”, “Dahl concept”, “occlusal veneers”, and “full-mouth rehabilitation” with emphasis on systematic reviews, longitudinal cohort studies, and randomized clinical trials. The review identified that VDO loss arises from a multifactorial etiological spectrum encompassing pathological tooth wear (erosion, attrition, abrasion, abfraction), tooth loss with posterior support collapse, parafunctional behaviors, iatrogenic factors, and systemic and pharmacological contributors. Population-based data indicate that the prevalence of severe tooth wear rises from approximately 3% at age 20 to 17% by age 70. Diagnosis remains predominantly empirical and integrative, triangulating anthropometric, phonetic, functional, and esthetic indicators - none of which is independently reliable. Digital diagnostic technologies offer measurable improvements in precision but remain insufficiently validated for standalone clinical use. Treatment philosophies span minimally invasive additive composite protocols (Dahl concept, three-step technique), indirect bonded ceramic rehabilitations (lithium disilicate, zirconia, ultrathin occlusal veneers), and full-arch tooth- or implant-supported reconstructions, with growing consensus around staged, reversible, and patient-centered decision-making. The contemporary management of loss of VDO requires a structured, individualized, and evidence-based approach. Clinical decisions should consider the underlying cause, the patient’s ability to adapt, functional and biomechanical needs, and a stepwise plan that starts with reversible procedures before moving to definitive treatment.

## Introduction and background

The vertical dimension of occlusion (VDO) is defined as the distance between two selected anatomical reference points (typically subnasale and gnathion, or marked points on the maxilla and mandible) when the teeth are in maximum intercuspation [1]. Maintaining VDO is crucial for orofacial harmony, efficient mastication, temporomandibular joint (TMJ) stability, clear phonetics, and the esthetic balance of the lower facial third. When VDO is disrupted, the effects extend well beyond the teeth themselves, influencing muscle function, soft-tissue support, joint loading, and ultimately the patient’s oral health-related quality of life [2].

Building on the foundations laid in Parts I and II of this series [2,3], this third part shifts focus to arguably the most challenging clinical scenario: managing patients who have lost their VDO, addressing the complexities when VDO is no longer preserved.

Rather than viewing VDO loss as a discrete event, it is more helpful to consider it a clinical syndrome - a multifactorial process with diverse causes, variable presentations, and treatment challenges that increase with severity, patient age, and the condition of the stomatognathic system [4,5]. Loss of VDO occurs on a spectrum. It can start with mild, often unnoticed changes that are compensated by natural tooth and bone adaptation, or progress to functional problems, and in severe cases, lead to major bite collapse with significant esthetic and functional difficulties. Identifying these stages and planning appropriate treatment requires careful diagnosis, understanding of biomechanics and biology, and a cautious, evidence-based approach that recognizes the limitations of current research [6].

Despite its clinical importance, research on VDO loss remains methodologically uneven [4,7]. High-quality randomized controlled trials are rare, diagnostic criteria lack consistency, and case definitions vary widely across studies. Furthermore, long-term outcome data are limited and largely based on retrospective case series with heterogeneous endpoints. Even the operational definition of what constitutes “significant” VDO loss has yet to be standardized. These shortcomings complicate cross-study comparisons, obscure evidence-based clinical boundaries, and force clinicians to navigate treatment decisions with a blend of solid biological reasoning, expert consensus, and inferential judgment.

This review aims to synthesize the contemporary literature on VDO loss. Specifically, it will (i) characterize the etiological factors leading to VDO loss, (ii) evaluate diagnostic methods for identifying and quantifying VDO loss, (iii) outline current clinical protocols ranging from minimally invasive to comprehensive rehabilitation, (iv) propose a structured framework to guide clinical decision-making, and (v) highlight research gaps that should inform the field’s future directions.

## Review

Methods

This narrative review was developed following the Scale for the Assessment of Narrative Review Articles (SANRA) guidelines [8], which offer a structured approach to designing, conducting, and reporting non-systematic reviews.

Information Sources and Search Strategy

We conducted extensive searches across PubMed/MEDLINE, the Cochrane Library, Scopus, and Web of Science between 1 October 2025 and 31 October 2025, covering all records up to October 2025. In addition, reference lists from relevant articles were manually examined to identify further pertinent sources. The search strategy combined controlled vocabulary (Medical Subject Headings, or MeSH) with free-text terms organized into four thematic domains: (i) vertical dimension, (ii) etiology, (iii) diagnosis, and (iv) treatment. Boolean operators (AND/OR) were applied to link these domains effectively. The full search strategies and Boolean syntax for each database are detailed in the Appendices.

Eligibility Criteria

Priority was given to including: (i) systematic reviews and meta-analyses, (ii) randomized clinical trials, (iii) prospective cohort studies with at least three years of follow-up, (iv) international consensus statements, (v) historically seminal references that remain relevant today, and (vi) authoritative textbook chapters when peer-reviewed primary evidence was lacking. Case reports and case series were incorporated only if they presented clinically significant techniques or illustrated areas of unresolved debate. Articles written in languages other than English, narrative editorials, and opinion pieces without supporting evidence were excluded.

Synthesis Approach

Each source was qualitatively assessed based on study design, sample size, follow-up duration, methodological transparency, and alignment with the broader evidence base. Instead of performing a statistical meta-analysis, the findings were combined using a thematic approach. The review highlights areas of agreement, disagreement, and gaps in the literature.

Limitations of the Review Methodology

Being a narrative rather than a systematic review, this synthesis is inherently vulnerable to selection bias, and no quantitative pooling of outcomes was performed. The intent is to offer a critical, pragmatic clinical overview, so conclusions should be interpreted within this context. Any statements regarding relative magnitudes, such as approximate failure rates, reflect predominant findings from the cited literature rather than formal meta-analytic estimates.

Etiology of VDO loss

**Figure 1:**
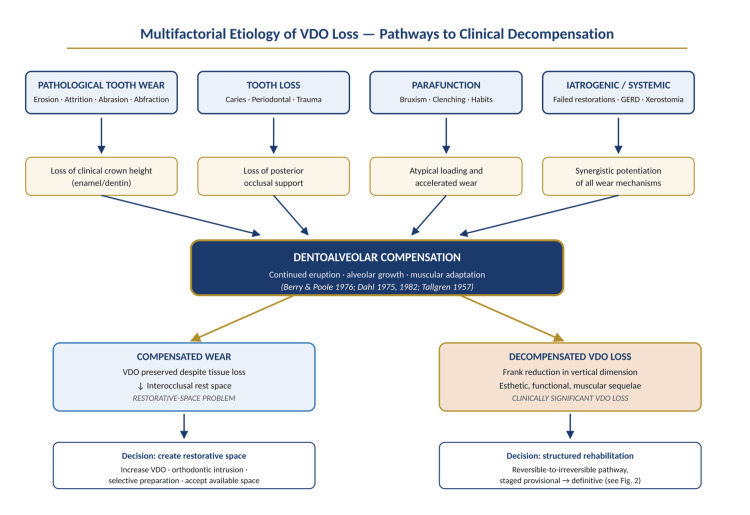
Multifactorial etiology of VDO loss. Four primary etiological domains converge on common biomechanical mechanisms that are mediated by dentoalveolar compensation. The clinical outcome determines whether the dominant clinical issue is the restorative-space problem or genuine VDO reconstruction. VDO: vertical dimension of occlusion; GERD: gastroesophageal reflux disease

The causes of loss of VDO are usually multifactorial. In most patients, VDO loss does not result from a single cause but rather from several factors acting together or over time during the patient’s life. Understanding these causes is important for accurate diagnosis, treatment planning, and predicting prognosis. Failure to identify and manage the underlying causes before definitive rehabilitation may increase the risk of restorative treatment failure [4,5]. Figure 1 illustrates the main etiological domains and highlights how various mechanisms converge on dentoalveolar adaptation, which ultimately determines whether the clinical outcome is compensated wear or decompensated VDO loss.

Tooth Wear: The Dominant Contemporary Driver

Pathological tooth wear - which includes erosion, attrition, abrasion, and abfraction - has become the principal pathway through which VDO is progressively lost in dentate populations worldwide [9,10]. A systematic review by Van’t Spijker et al. reported that the prevalence of severe tooth wear increases markedly with age, from around 3% at 20 years old to 17% by age 70, demonstrating significant age-related progression [9]. These wear mechanisms rarely act in isolation; rather, they interact synergistically, producing tissue loss far greater than any single process could cause alone [4,11]. Contemporary classifications such as the Tooth Wear Evaluation System (TWES) [11] and the Basic Erosive Wear Examination (BEWE) [12] have improved the standardization of assessment, yet they remain underutilized in routine restorative practice.

Erosion

Dental erosion - defined as the chemical, non-bacterial dissolution of dental hard tissues - now stands out as the leading driver of tooth wear, especially among younger and middle-aged adults [10,13]. Both intrinsic and extrinsic acid sources play roles. Intrinsic erosion typically results from gastroesophageal reflux disease (GERD), eating disorders such as bulimia nervosa, chronic regurgitation, and rumination. It characteristically affects the palatal surfaces of maxillary anterior teeth and the occlusal surfaces of posterior teeth [10]. Because early erosion is often painless and subtle in appearance, it frequently advances significantly before being noticed by patients or clinicians.

Extrinsic erosion arises primarily from dietary acid exposure and, less commonly, occupational or environmental acid contact [10,13]. Epidemiological studies over the last 30 years document a steady rise in erosive tooth wear, especially in younger populations [13,14]. However, individual susceptibility varies widely, influenced by factors such as salivary buffering capacity, flow rate, fluoride availability, enamel composition, and tooth morphology [10].

Clinically, erosion-driven VDO loss is challenging due to its tendency for bilateral, generalized, and biomechanically indiscriminate distribution [15,16]. Severe erosion often involves all dental segments simultaneously, complicating reconstruction by removing stable reference points for VDO and making staged rehabilitation more difficult [15].

Attrition

Attrition - wear resulting from direct tooth-to-tooth contact - is a lifelong, physiological process. However, when accelerated pathologically, it can cause clinically significant VDO reduction within surprisingly short periods [4]. Bruxism, both nocturnal and diurnal, is the most common culprit. Parafunctional attrition produces characteristic flat, polished, and congruent wear facets, which contrast with the cupped morphology typical of erosion [4,11].

The role of bruxism in VDO loss warrants careful consideration. The 2018 international consensus redefined bruxism as repetitive masticatory muscle activity rather than a disorder per se, emphasizing that its clinical significance depends on consequences such as tooth wear, muscle hypertrophy, and TMJ overload [17]. Although it seems intuitive that bruxism causes VDO loss, the relationship is mechanistically complex. Many individuals with bruxism maintain dentoalveolar compensation for years, preserving clinical VDO until a point of decompensation is reached [6]. The exact threshold and timing of this transition remain poorly understood.

Abrasion and Abfraction

Abrasion results from external mechanical processes and mainly contributes to VDO loss through cervical tissue destruction, although severe generalized abrasion can also reduce occlusal height [11]. Abfraction involves cervical microfractures due to occlusal stress, presenting typically as cervical notches rather than crown shortening; its independent role in VDO loss is limited. Nevertheless, these processes deserve attention because they often coexist with erosion and attrition, accelerating overall tissue loss [11].

The biological interplay among wear mechanisms is crucial. Acidic environments soften enamel surfaces, significantly reducing their resistance to mechanical wear from attrition or abrasion [10]. Laboratory studies support that the combined mechanical-erosive assault accelerates tissue loss far beyond what either mechanism causes alone [10]. This interaction highlights the inadequacy of attributing tooth wear to a single cause and stresses the importance of comprehensive, multidimensional etiological assessment [4,11].

Tooth Loss and Posterior Support Collapse

Posterior dental support loss - caused by caries, periodontal disease, trauma, or extraction - triggers a biomechanical cascade that, if left uncorrected, can lead to progressive reduction in VDO [18,19]. Posterior teeth bear the majority of occlusal load during mastication and serve as the “pillars” of occlusal stability. Their absence shifts the load onto anterior teeth, which are biomechanically ill-equipped to handle such forces. This may result in combination syndrome (or anterior hyperfunction syndrome), especially in maxillary edentulous patients opposing mandibular natural anterior teeth, characterized by anterior maxillary alveolar bone resorption, accelerated anterior tooth wear, papillary hyperplasia, and progressive mandibular overclosure [18]. Even in patients with some dentition, losing bilateral posterior support can produce a similar but less pronounced pattern.

However, the timeline between posterior tooth loss and VDO reduction is neither linear nor consistent. Compensatory eruption, dentoalveolar adaptation, and the viscoelastic properties of periodontal tissues can maintain VDO for variable periods after tooth loss [6,19]. The rate and extent of collapse depend on factors such as the number and distribution of missing teeth, symmetry of loss, presence of parafunctional habits, and biomechanical adequacy of remaining dentition. This variability complicates diagnosis, as radiographic or clinical signs of posterior support loss do not reliably reflect VDO compromise.

Parafunctional Habits

Beyond bruxism, a broader range of parafunctional behaviors contributes to VDO loss by accelerating wear and imposing atypical loading patterns. These include clenching, pipe or pencil biting, nail-biting, lip and cheek biting, and certain occupational habits [17]. Identifying such behaviors is vital because their persistence during or after rehabilitation significantly raises the risk of prosthetic failure. Evidence increasingly links psychosocial stress with heightened bruxism intensity and parafunctional activity, although the directionality and effect size remain debated [17]. Clinically, this suggests that comprehensive VDO rehabilitation often benefits from a multidisciplinary approach incorporating behavioral and psychological management, especially in patients exhibiting severe or progressive wear [4].

Iatrogenic and Restorative Factors

Iatrogenic factors play a clinically significant yet often underrecognized role in VDO loss. Poorly contoured restorations, inadequate occlusal contacts, premature wear, and the cumulative effect of repeatedly replacing failing restorations without addressing underlying wear can destabilize occlusion and progressively reduce VDO [20]. Excessive tooth reduction during preparation also compromises VDO; pioneering biomechanical research by Edelhoff and Sorensen showed that conventional crown preparations remove substantial coronal volume, adversely affecting pulp vitality and long-term restoration prognosis [20,21]. Moreover, long-term use of inadequately maintained removable partial dentures lacking posterior occlusal support, or chronically underrelined complete dentures associated with progressive denture base resorption, can cause gradual but clinically significant VDO loss that often goes unnoticed initially [22].

Systemic, Pharmacological, and Developmental Contributors

Systemic conditions can predispose individuals to VDO loss through various pathways. Sjögren’s syndrome and other xerostomia-inducing diseases accelerate erosive and cariogenic destruction by eliminating saliva’s buffering and remineralizing effects [10]. GERD and bulimia nervosa are particularly noteworthy, as their dental manifestations often precede medical diagnosis [10,13]. Pharmacological agents - including xerostomic medications, acidic formulations, and drugs causing erosive gastric symptoms - further exacerbate the wear environment. Developmental disorders such as amelogenesis imperfecta and dentinogenesis imperfecta result in structurally compromised enamel and dentin, rendering teeth highly susceptible to wear and leading to early-onset, severe VDO loss.

The intersection of systemic and pharmacological factors with local wear mechanisms underscores why a thorough medical history, dietary assessment, and medication review are indispensable parts of the diagnostic process [4]. Missing these systemic contributors before initiating definitive restorative treatment is a well-recognized cause of early failure [4]. Table 1 summarizes the etiological categories of VDO loss and principal clinical features.

**Table 1:** Etiological categories of VDO loss and principal clinical features. VDO: vertical dimension of occlusion

Etiological Category	Principal Mechanism	Characteristic Clinical Features
Erosion	Chemical dissolution (intrinsic/extrinsic acid)	Cupped occlusal surfaces; smooth, glazed appearance; palatal involvement; restorations standing proud of adjacent enamel [10,12]
Attrition/bruxism	Tooth-to-tooth contact under parafunctional load	Flat, polished, congruent wear facets; muscle hypertrophy; cracked tooth syndrome [11,17]
Abrasion	External mechanical wear (brush, abrasive dentifrice, occupational)	Cervical V-shaped notches; localized to accessible surfaces
Abfraction	Stress-induced cervical microfracture	Wedge-shaped cervical lesions; may coexist with erosion
Posterior tooth loss	Loss of biomechanical occlusal support	Anterior over-eruption; over-closure; combination syndrome features [18]
Iatrogenic	Failing restorations, over-reduction, inadequate occlusal scheme	Asymmetric VDO loss; instability of occlusal contacts; localized wear [20,21]
Systemic/pharmacological	Xerostomia; reflux; medication side-effects	Generalized rapid wear; cervical caries; mucosal changes [10]
Developmental	Defective enamel/dentin formation	Early-onset, severe wear with abnormal tooth morphology

Critical Appraisal: The Etiological Evidence Base

Most of the available evidence on the causes of VDO loss comes from cross-sectional studies, expert opinions, and case reports or case series. There are very few long-term prospective studies that measure the rate of VDO loss or evaluate the exact contribution of different causes in individual patients. Although the BEWE [12] and TWES [11] indices have improved tooth wear assessment standardization, they have rarely been used to track VDO changes prospectively. Future research should prioritize cohort designs combining digital wear monitoring with longitudinal VDO measurement to fill this gap.

Compensatory mechanisms of VDO loss

Among the most clinically significant consequences of VDO loss is dentoalveolar compensation - the stomatognathic system’s remarkable tendency to maintain vertical facial dimensions via adaptive remodeling, even amid substantial tissue loss [6,23]. This compensatory mechanism has a profound impact on diagnosis, treatment planning, and prognosis, making it essential to consider carefully before deciding on VDO restoration.

Dentoalveolar Adaptation

The classical framework established by Dahl et al. [23], supported by later clinical and cephalometric studies [6,24,25], highlights the alveolar process’s impressive ability to adapt to altered occlusal forces and tooth positions through continuous eruption and bone remodeling. Berry and Poole further proposed that a combination of secondary dentine formation, alveolar growth, and adaptive changes in the masticatory muscles forms an integrated compensatory system that sustains masticatory function even in the face of extensive attrition [6]. In cases of progressive tooth wear, this means that the clinical crown height - and thus the clinical VDO - may remain surprisingly stable despite significant loss of tooth substance [25]. Essentially, compensatory eruption offsets crown height reduction, preserving occlusal contacts but at the cost of increased alveolar bone height and a diminished interocclusal rest space (IRS) [6].

This has important clinical implications that are often overlooked: loss of tooth structure does not necessarily equate to loss of VDO. Patients with severe wear may still present with near-normal VDO and a notably reduced freeway space, rather than the collapsed, over-closed appearance one might anticipate [25]. This distinction directly affects treatment planning, particularly regarding restorative space availability. When compensation is fully established, gaining sufficient restorative space involves either orthodontic intrusion of the worn teeth, deliberately increasing VDO beyond the compensated level, or accepting restorations of minimal thickness [4,6].

The Spectrum of Compensation

Compensation is rarely an all-or-nothing phenomenon. Clinical cases exist along a spectrum, ranging from complete compensation, where VDO is maintained despite severe tooth wear, to partial compensation, where some reduction in VDO occurs but is less than expected, and finally to no compensation, where clear clinical loss of VDO is present [6,24,25]. The amount of compensation depends on the balance between the rate of tooth wear and the ability of the teeth and surrounding structures to adapt. This adaptive capacity can be affected by factors such as age, periodontal condition, parafunctional habits, and systemic health conditions [6].

Distinguishing these states can be challenging. A patient who appears over-closed might truly have lost VDO or may simply have a facial morphology that mimics over-closure. Conversely, patients with apparently normal facial proportions might have compensated for considerable VDO loss, masking an underlying problem [3,25]. This diagnostic uncertainty underscores the necessity of integrating multiple diagnostic criteria rather than relying on any single clinical sign.

The Restorative Space Problem

Compensation introduces perhaps the most pressing practical challenge in managing worn dentitions: the restorative space problem [4,6]. When wear occurs without significant compensation, the resulting biological increase in IRS naturally allows for restorative materials to be placed. However, when compensation has taken place, available rest space can be severely limited-sometimes to the extent that providing adequate restorative thickness without altering VDO becomes impossible [4,16].

Addressing this issue demands a clear clinical decision among four main strategies: (i) accepting the limited space and choosing restorative materials accordingly; (ii) creating space through selective tooth preparation - often a technically demanding approach given the already compromised tooth structure [21]; (iii) increasing VDO to generate space above the compensated occlusal plane [23,26]; or (iv) employing orthodontic intrusion to gain space without changing VDO. Each approach carries unique biomechanical, biological, and patient-centered considerations that must be carefully weighed [4].

Clinical diagnosis of VDO loss

Diagnosing loss of VDO remains a challenging clinical puzzle. There isn’t a single, objective measure that reliably quantifies VDO reduction across patients [3,7]. Since most individuals lack a documented “original” VDO, and many clinical signs are inherently subjective, clinicians must rely heavily on their judgment, synthesizing various imperfect clues to reach a reasoned conclusion [3].

Clinical Signs and Symptoms

The clinical manifestations of VDO loss are broad, affecting soft tissues, dental structures, and the musculoskeletal system. No single sign definitively diagnoses the condition; rather, it’s the overall pattern of findings, interpreted alongside the patient’s history, habits, and functional concerns, that guides diagnosis [2,3].

Soft-Tissue and Esthetic Manifestations

Facial esthetics often provide some of the most telling clues. Progressive overclosure alters the lower third of the face, leading to deepened labiomental folds, a more prominent chin relative to the lips, accentuated nasolabial folds, and a forward-downward mandibular rotation. Patients may exhibit lip incompetence or compromised lip support and, in advanced stages, develop angular cheilitis [2]. However, angular cheilitis, while classic, is neither universal nor specific to VDO loss.

Interpreting these changes calls for caution. Age-related soft tissue laxity and fat redistribution can mimic some of these features [24]. Moreover, skeletal patterns influence appearance: for instance, a Class III jaw relationship or a prominent chin can exaggerate perceived overclosure, whereas a Class II pattern may mask it. Thus, facial esthetic signs are best viewed as supportive rather than definitive diagnostic markers [3].

Masticatory Muscle and TMJ Findings

Altering the vertical dimension affects the masticatory muscles and TMJs. With VDO loss, muscles such as the masseter and temporalis work at changed insertion angles and shorter resting lengths, which can lead to fatigue, tenderness, or adaptive hypertrophy [2].

The link between VDO loss and temporomandibular disorders (TMDs) is complex and needs careful consideration. Earlier models emphasizing occlusion as a primary cause of TMD have been challenged by recent evidence [27,28]. Manfredini et al.’s systematic review found no strong support for occlusion as a main factor in TMD pathophysiology and recommended moving away from traditional gnathological views in TMD management [28]. According to the Diagnostic Criteria for Temporomandibular Disorders (DC/TMD), TMD is a multifactorial biopsychosocial condition, with occlusion playing at most a secondary role [27]. Clinically, this means irreversible occlusal changes, including VDO alterations, should not be performed solely to treat TMD unless there is a clear, independent prosthodontic indication [27,28].

Phonetic Assessment

Phonetic evaluation provides a functionally relevant, somewhat reproducible adjunct to VDO assessment [2,3]. Speech sounds, especially fricatives and sibilants, depend on precise spatial relationships between dental arches. The “closest speaking space” concept suggests that during sibilant articulation, a 1-3 mm space between incisal edges indicates an appropriate VDO range [3].

Despite its usefulness, phonetic assessment has limitations. Interpersonal variability in speech patterns is considerable, and patients often adapt over time to altered VDO, diminishing the diagnostic value of phonetics alone. Therefore, it’s best employed as a corroborative tool rather than a primary determinant [3].

Anthropometric and Cephalometric Assessment

Classical anthropometric methods, such as comparing facial thirds or using Willis gauge facial height ratios, are commonly taught but have important diagnostic limitations [3]. The “equal thirds” principle is based on population averages and does not reliably capture individual anatomical variation [3,24].

Cephalometric analysis becomes particularly valuable in complex cases [24,25]. Tallgren’s seminal longitudinal studies documented systematic face height changes related to tooth wear, loss, and prosthetic interventions, laying the groundwork for modern understanding [24]. Cephalometry offers quantitative data on lower facial height, mandibular plane angle, condylar position, and incisor relationships. Its primary role is in multidisciplinary cases involving orthodontic-prosthodontic or surgical-prosthodontic collaboration [3].

IRS and the Physiological Rest Position

The IRS, or freeway space - the gap between maxillary and mandibular teeth when the jaw is at physiological rest - remains a central clinical parameter [2,3]. Normatively, an IRS of 2-4 mm in the molar region serves as a useful reference. An IRS significantly exceeding this, especially alongside other signs of VDO loss, supports a diagnosis of true vertical dimension reduction. Conversely, a markedly reduced IRS in the presence of extensive tooth wear suggests a compensatory adaptation that impacts restorative planning [6].

However, assessing IRS reliably hinges on accurately determining the physiological rest position - a task fraught with methodological challenges, as discussed in Parts I and II of this series [2,3]. Factors such as head posture, emotional state, muscle fatigue, and technique influence rest position variability. Although recent kinesiographic technologies improve measurement reproducibility, they have yet to demonstrate clear superiority over meticulous conventional clinical assessment [3].

Mounted Study Casts and the Diagnostic Wax-Up

Mounting diagnostic casts on semi- or fully adjustable articulators, with the mandible recorded at centric relation or a verified intercuspal position, provides an indispensable three-dimensional perspective [3,4,15]. This approach allows visualization of occlusal relationships, wear patterns, and available interocclusal space in ways that intraoral examination alone cannot achieve. Creating a diagnostic wax-up forms a working hypothesis of the patient’s optimal occlusal scheme, which should ideally be tested through provisional restorations before definitive treatment [15,16,29].

Accuracy in facebow transfer and centric relation records is critical; errors here can create false impressions of VDO discrepancies that don’t exist clinically. Hence, standardized protocols are essential to ensure reliable articulator-based evaluation [3].

Digital and Technological Diagnostic Adjuncts

Digital technologies are increasingly shaping diagnostic capabilities in VDO assessment [3]. Tools such as three-dimensional intraoral and extraoral scanners, digital radiography, cone-beam computed tomography (CBCT), jaw motion tracking, and surface profilometry offer enhanced precision, reproducibility, and the ability to document changes longitudinally compared to traditional methods [3,30,31].

Intraoral scanning paired with virtual articulator software enables detailed 3D mapping of wear and precise virtual assessment of interocclusal relationships, often outperforming conventional impressions in accuracy [30,31]. Serial scans taken over time allow clinicians to quantify tooth wear progression, a feature with significant clinical and research implications [11,30].

Nonetheless, it’s important to distinguish technical precision from clinical validity. While many digital systems provide impressive reproducibility, robust evidence demonstrating that digital VDO measurements translate into better patient outcomes than conventional approaches remains lacking [3]. Table 2 summarizes the reliability and clinical utility of diagnostic parameters for VDO loss.

**Table 2:** Diagnostic parameters for VDO loss. VDO: vertical dimension of occlusion; TWES: Tooth Wear Evaluation System; BEWE: Basic Erosive Wear Examination

Parameter	Reliability	Clinical Utility	Evidence Base
Facial esthetic/soft-tissue assessment	Moderate; influenced by age and skeletal pattern	High as adjunct; insufficient alone	Expert opinion/case series [2]
Anthropometric facial thirds	Low; significant individual variability	Limited; corroborative only	Expert opinion [3]
Interocclusal rest space (IRS)	Moderate; method-dependent	High when combined with other findings	Clinical studies [3]
Phonetic assessment (closest speaking space)	Moderate; adaptive masking possible	Useful corroborative tool	Clinical studies [3]
Articulator-mounted casts and wax-up	High when records are accurate	Essential for treatment planning	Consensus/clinical evidence [4,15]
Cephalometric analysis	High for structural measurement	Selective use in complex cases	Longitudinal studies [24]
Intraoral 3D scanning + virtual articulation	High precision	Growing role; useful for monitoring	Emerging evidence [30,31]
Tooth wear indices (BEWE, TWES)	Acceptable to good (validated)	Diagnostic standardization, monitoring	Validated indices [11,12]
Stereophotogrammetry/3D facial imaging	High	Documentation; outcome assessment	Emerging evidence [3]
Jaw motion tracking (kinesiography)	Variable	Limited; insufficient clinical validation	Limited clinical studies [3]

Critical Appraisal: Diagnostic Evidence

To date, no diagnostic parameter for VDO loss has been validated against an objective gold standard. Most recommendations derive from expert consensus, classical clinical experience, and cross-sectional studies. The lack of operational diagnostic criteria - similar to the DC/TMD system for TMDs [27] - represents a fundamental limitation, impeding both clinical research and quality improvement efforts.

Clinical decision-making framework

**Figure 2:**
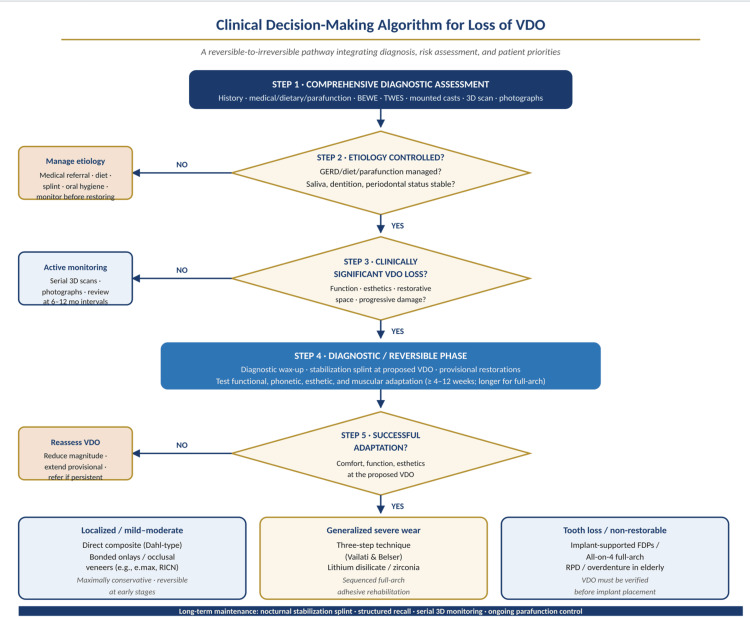
Clinical decision-making algorithm for loss of VDO. The algorithm progresses from comprehensive diagnostic assessment (Step 1) through etiological control (Step 2), clinical significance triage (Step 3), and a mandatory diagnostic-reversible phase (Step 4), to verification of patient adaptation (Step 5) before definitive rehabilitation is selected (Step 6). The pathway emphasizes the reversible-to-irreversible principle and integrates current consensus on minimally invasive management. VDO: vertical dimension of occlusion; TWES: Tooth Wear Evaluation System; BEWE: Basic Erosive Wear Examination; GERD: gastroesophageal reflux disease; FDPs: fixed dental prostheses; RPD: removable partial dentures; RICN: Resin Infiltrated Ceramic Network

Deciding whether to restore the VDO, determining the extent of change, and selecting the appropriate method requires an integration of diagnostic data, individual patient factors, biological principles, and risk assessment into a tailored, evidence-informed treatment plan [4]. Since many consequences of this decision are irreversible, it demands a structured approach rather than relying on habitual techniques. Figure 2 outlines a stepwise clinical decision-making algorithm - beginning with comprehensive diagnostics, progressing through etiological control and clinical significance assessment, advancing to a reversible diagnostic phase, and culminating in the selection of definitive treatment - that embodies the principles discussed below.

Is VDO Loss Clinically Significant?

One of the most critical initial questions is whether the observed tooth wear or altered occlusal relationships genuinely represent clinically significant VDO loss that warrants intervention, or if they are merely physiological adaptations that do not [4,6]. As mentioned earlier, compensatory dentoalveolar changes often preserve VDO despite substantial tissue loss. Therefore, the mere presence of worn dentition alone does not automatically justify restoring the VDO.

Clinically significant VDO loss can be pragmatically defined as a reduction sufficient to cause one or more of the following: functional impairment (such as compromised mastication, speech difficulties, or muscular dysfunction); esthetic concerns deemed unacceptable by the patient; inadequate restorative space to accommodate durable and biomechanically sound restorations; or ongoing progressive tissue destruction that threatens the long-term health of the dentition [4]. The presence of any of these factors strengthens the indication for intervention, whereas their absence - especially in a stable, asymptomatic patient - may favor active monitoring rather than immediate treatment [4].

The Reversible-to-Irreversible Continuum

A key guiding principle is the careful progression from reversible to irreversible treatment modalities [4,15]. Reversible methods - such as diagnostic occlusal splints, provisional composite restorations, and removable appliances - offer the opportunity to assess the patient’s functional and adaptive response to a proposed change in VDO before committing to permanent tooth preparation [16,29]. This approach reflects more than just procedural caution; it acknowledges the biological reality that predicting patient adaptation to VDO alterations cannot be reliably achieved through diagnostics alone [2,6].

Therapeutic-diagnostic splints, constructed at the proposed new VDO, serve a dual purpose: they both test adaptability and provide symptomatic relief from parafunctional loading [27]. Successful patient adaptation - typically evaluated over a period ranging from several weeks to months, depending on case complexity - offers reasonable confidence that the intended VDO change falls within the patient’s adaptive capacity [4]. Conversely, persistent muscle pain, speech difficulties, or TMJ symptoms indicate the need for reassessment. It is important to note, however, that adaptation to a splint does not guarantee similar tolerance to definitive restorations, which differ in occlusal contacts, material properties, and geometry [3,15].

How Much VDO Should Be Restored?

Determining the precise amount of VDO increase remains a deceptively complex issue that has sparked considerable debate [4,15]. Options range from minimal increases - just enough to accommodate restorative materials - to more substantial adjustments aimed at restoring the patient’s presumed original VDO or addressing esthetic concerns.

Since verifiable historical VDO data are rarely available, clinicians must rely on diagnostic estimations using methods reviewed earlier in this series [3], none of which has demonstrated clear superiority. A practical approach, supported by various authors and reflected in the European consensus on severe tooth wear, is to identify the minimum VDO increase needed to resolve the clinical problems and adopt this functional minimum as the treatment goal [4,15]. Any increase beyond this should be specifically justified and tested through provisional restorations before finalizing treatment. This conservative strategy aligns with biological prudence and the existing evidence on patient adaptation [4,29].

Staged Versus Simultaneous Rehabilitation

Current and available evidence strongly favors a staged rehabilitation approach rather than a single-phase, simultaneous full-arch reconstruction [4,15,16]. The staged method involves placing provisional restorations at the proposed VDO and maintaining them for a defined period before definitive restorations are fabricated. This strategy offers several benefits: it allows assessment of patient adaptability, provides the patient with a chance to evaluate esthetic outcomes and request adjustments, facilitates progressive refinement of the occlusal scheme, and helps identify unforeseen functional issues before irreversible treatment is completed [15,16].

The optimal duration of the provisional phase is not firmly established by high-level evidence. Recommendations vary from several weeks to several months and generally lengthen with increasing case complexity [4,15]. Notably, some patients may choose to retain durable provisional restorations for extended periods or even indefinitely, underscoring that the provisional phase serves as a genuine therapeutic and diagnostic interval rather than just a temporary waiting period.

Anterior-First Versus Posterior-First Sequencing

The sequencing of arch and segment restoration has long been a topic of philosophical debate. Two main approaches dominate: the anterior-first approach, popularized by Dawson, establishes anterior guidance and incisal relationships first, then builds the posterior occlusion to harmonize with this reference; conversely, the posterior-first approach sets the posterior occlusal scheme and VDO before finalizing anterior relationships [32]. The Vailati-Belser three-step technique offers a streamlined, anterior-led adhesive workflow specifically tailored for severely eroded dentitions [15,16].

Each approach presents distinct rationales and technical challenges. The choice often depends on the clinical scenario - wear distribution, individual tooth condition, and restorative complexity - rather than a one-size-fits-all preference [4,15]. Hybrid strategies, restoring segments in a planned sequence according to specific clinical priorities, are common in complex cases. Importantly, the literature does not support the categorical superiority of either philosophy, and rigid adherence to one method is unwarranted.

Critical Appraisal: Decision-Making Evidence

The framework guiding VDO restoration decisions rests largely on expert consensus [4], retrospective case series, and observational cohort studies rather than prospective randomized trials. Variability in case definitions, outcome measures, and follow-up durations further complicates cross-study comparisons. The widespread use of diagnostic splint therapy as a prerequisite to VDO changes, while clinically sensible, has not been rigorously compared with alternative pathways. The field would greatly benefit from prospective cohort studies employing standardized criteria, validated outcomes, and sufficient follow-up periods.

Clinical protocols for VDO restoration

The rehabilitation of the lost VDO spans a spectrum of clinical strategies. These range from minimally invasive additive composite techniques through indirect bonded ceramic restorations to full-arch tooth- or implant-supported reconstructions. Choosing the appropriate method hinges on multiple factors: the cause and severity of VDO loss, the pattern and extent of tooth wear or loss, the patient’s functional and esthetic demands, as well as the clinician’s expertise and available resources [4,15].

The Dahl Concept and Modern Additive Composite Protocols

Originally described by Dahl, Krogstad, and Karlsen in 1975 [23], the Dahl concept offers one of the most conservative options for restoring VDO when conditions permit. The initial approach involved a removable cobalt-chromium anterior platform that increased the anterior vertical dimension, allowing posterior teeth - initially left out of occlusion - to erupt gradually into contact. This approach protected and rebuilt the worn anterior teeth. Cephalometric studies later confirmed that the increased face height resulted from a combination of intrusion of the loaded teeth and eruption of the unloaded ones, reliably re-establishing occlusal contacts [23,25,26]. More recently, this principle has been adapted to direct composite resin restorations bonded to worn teeth at an elevated VDO [26,33].

Today’s composite-based Dahl approach presents several advantages: it is reversible in early stages, preserves tooth structure, and can be integrated seamlessly into routine restorative workflows. Systematic reviews have reported favorable outcomes in carefully selected patients [33,34]. For instance, a 10-year follow-up study by Gulamali et al. showed acceptable survival rates for composite Dahl restorations, with manageable maintenance requirements [34]. The European consensus on severe tooth wear supports additive composite restorations as an appropriate initial treatment in selected cases [4].

That said, the technique has its limitations. Patient selection remains crucial. It is best suited for younger individuals with sufficient adaptive capacity, mild to moderate localized anterior wear, and intact posterior support [33]. In cases of severe generalized wear, significant posterior support loss, or reduced adaptability, posterior re-establishment may be incomplete or unpredictable. Additionally, the long-term durability of direct composite in high-load environments is a concern; reported annual failure rates typically lie in the low single-digit percentages, varying by patient population and study criteria [33,34]. Therefore, repeated maintenance, repairs, and eventual replacement should be anticipated [4].

The Vailati-Belser Three-Step Technique

For patients exhibiting generalized erosive tooth wear coupled with significant VDO loss, the three-step technique developed by Vailati and Belser [15,16] has gained traction as a structured, minimally invasive protocol. This approach proceeds in phases: (i) maxillary anterior facial reconstruction via a vestibular approach to set new tooth length and esthetic plane, (ii) posterior rebuild at the new VDO using palatal or occlusal composite or ceramic restorations to establish a stable posterior stop, and (iii) maxillary anterior palatal reconstruction to finalize anterior guidance and complete the rehabilitation [15,16]. This method exemplifies sequenced reconstruction with validation of VDO changes through provisional phases.

Mid-term follow-ups of patients treated with the three-step technique report satisfactory survival of bonded restorations alongside positive patient-reported outcomes. However, long-term data beyond 5 to 10 years remain scarce [15,16]. The protocol demands considerable clinical and laboratory skill and achieves best results when combined with careful case selection, effective control of parafunctional habits, and structured maintenance, including nocturnal stabilization splints [4,15].

Indirect Restorative Protocols

When patients present with moderate-to-severe VDO loss, generalized wear, or insufficient tooth structure for direct bonding, indirect restorations using ceramics, ceramic-composite hybrids, or, less commonly nowadays, metal-ceramic restorations offer enhanced durability, precision, and esthetic outcomes [21,35].

Lithium Disilicate and Zirconia Ceramics

Lithium disilicate and zirconia ceramics have largely replaced metal-ceramic restorations in many VDO rehabilitation scenarios, driven by their superior esthetic qualities and, in the case of modern zirconia, exceptional mechanical properties [35,36]. Lithium disilicate allows for full-contour designs with excellent optical characteristics and can be bonded to minimally prepared or even unprepared tooth surfaces. This is particularly advantageous in worn dentitions where preserving remaining tooth structure is critical [21,35]. Systematic reviews indicate five-year survival rates comparable to metal-ceramic prostheses for multi-unit fixed dental prostheses, though studies extending beyond five years are still limited [35,36].

Zirconia’s high fracture toughness and flexural strength make it the material of choice for posterior regions subjected to heavy occlusal loading and for patients with bruxism [35,36]. However, its hardness relative to enamel raises concerns about antagonist tooth wear. Earlier generations of zirconia were also limited by opacity, restricting their esthetic versatility. Recent translucent, multi-layered zirconia formulations have addressed these esthetic shortcomings, although long-term data on wear behavior are still emerging [35]. Ultimately, the decision between lithium disilicate and zirconia should be individualized based on patient-specific factors.

Posterior Onlays, Occlusal Veneers, and Polymer-Ceramic Hybrids

Minimally invasive indirect restorations, such as ceramic onlays, occlusal veneers, and resin-infiltrated ceramic networks, occupy an important middle ground between direct composites and full-coverage crowns [37,38]. The Schlichting-Magne protocol for ultrathin (0.4-1.3 mm) computer-aided design/computer-aided manufacturing (CAD-CAM) occlusal veneers is a technically demanding yet conservative option backed by randomized clinical trial data with follow-up of up to three years [37,38].

In a prospective randomized trial by Schlichting et al., ultrathin lithium disilicate (e.max CAD) and resin-composite (Lava Ultimate) occlusal veneers demonstrated statistically similar mid-term clinical performance. Notably, restorable chipping was more common in the resin-composite group, which also exhibited higher surface degradation [38]. Across the literature, annual failure rates for occlusal veneers and posterior onlays generally remain in the low single digits, with ceramic fractures being the most frequent failure mode in patients with parafunctional habits [37]. Careful occlusal management, minimizing lateral working contacts on ceramic surfaces and ensuring sufficient material thickness, consistently emerges as key to longevity [4,38].

Full-Arch Tooth-Supported Rehabilitation

When natural teeth remain restorable, but the patient suffers from severe, generalized wear or tooth loss affecting multiple segments, full-arch tooth-supported prosthetic rehabilitation represents the most complex and technically demanding approach to restoring VDO [32,35]. Success requires meticulous planning, precise execution, and a deep understanding of occlusal principles at the reconstructed dimension.

The choice of occlusal scheme has been widely debated. Mutually protected occlusion is the most commonly recommended scheme in fully reconstructed dentitions [32]. While its biomechanical rationale and protective advantages are clear, evidence supporting its superiority in long-term prosthetic survival is limited. Moreover, individual patient factors often necessitate pragmatic adaptations to this theoretical ideal.

Long-term survival data for full-arch tooth-supported VDO restorations are constrained by heterogeneity in case definitions, restorative materials, and outcome reporting [35,36]. Nevertheless, available studies suggest acceptable long-term outcomes in carefully selected patients. Complication rates tend to be higher than for simpler treatments but are generally considered acceptable given the treatment’s complexity. Common issues include ceramic fractures, retention loss, periodontal complications, and endodontic problems in teeth with prior pulp involvement [35].

Implant-Supported Rehabilitation in VDO Loss

Dental implants have revolutionized prosthetic options for patients experiencing VDO loss due to tooth loss by offering fixed solutions that avoid preparation of remaining natural teeth [39,40]. Pjetursson et al. reported a five-year survival rate of about 95% and a 10-year survival of 80% for implant-supported fixed dental prostheses, with veneering material fractures and biological complications representing the chief adverse events [39].

Incorporating implants into VDO rehabilitation introduces unique challenges, notably the need to establish and confirm the definitive VDO before implant placement. Unlike natural teeth, osseointegrated implants are ankylosed and cannot adapt to subsequent vertical dimension changes once placed. Therefore, it is essential to validate the proposed VDO - ideally through a provisional phase - prior to implant surgery to ensure optimal prosthetic outcomes [3].

Full-arch implant-supported rehabilitation, including the All-on-4® concept and its variants, has become increasingly popular for edentulous or severely compromised patients [40]. A longitudinal study by Maló et al. following 471 patients over 10 to 18 years reported cumulative implant survival rates around 94-95%, with prosthetic success rates exceeding 99% in carefully selected cohorts [40]. Although these figures are encouraging, complications such as prosthetic fractures, screw loosening, and peri-implant disease remain clinically significant. The evidence base, while expanding, is largely drawn from retrospective and prospective observational studies conducted in specialized centers.

Occlusal Splints in Long-Term Management

Occlusal splints continue to play a vital role after VDO rehabilitation, especially for patients with confirmed parafunctional habits [17,27]. The use of nocturnal stabilization splints post-rehabilitation is widely recommended to protect against ceramic fractures, implant prosthesis overload, and accelerated wear of new restorations [4,15]. Although evidence supporting splints in preventing prosthetic complications stems mostly from clinical consensus and biomechanical reasoning rather than controlled trials, their biological plausibility and clinical rationale remain strong. The European consensus on severe tooth wear endorses splint use as an integral component of long-term maintenance [4].

However, patient compliance with long-term splint wear is variable. Fabrication and ongoing adjustment of well-fitting splints for patients with complex full-arch rehabilitations require continuous clinical attention. Incorporating splint therapy into comprehensive maintenance protocols, including regular recall visits, professional cleaning, radiographic monitoring, and periodic reassessment of occlusion, is essential for sustainable VDO management [4]. Table 3 summarizes clinical protocols for VDO restoration.

**Table 3:** Clinical protocols for VDO restoration. VDO: vertical dimension of occlusion; FDPs: fixed dental prostheses; CAD-CAM: computer-aided design/computer-aided manufacturing

Protocol	Best Indication	Advantages/Limitations	Key Evidence
Direct composite (Dahl-type)	Localized anterior wear; younger patients with adaptive potential	Reversible, conservative; limited durability under heavy load	[23,26,33,34]
Three-step technique (Vailati-Belser)	Generalized erosive wear; severe but restorable dentition	Structured, conservative, sequenced; technically demanding	[15,16]
Ultrathin CAD-CAM occlusal veneers	Posterior eroded wear; preservation of structure	Bonded, conservative, esthetic; sensitive to thickness	[37,38]
Lithium disilicate full-coverage	Moderate-severe wear; esthetic priority	Excellent esthetics; bondable; limited reserve in heavy bruxers	[35,36]
Translucent zirconia full-coverage	Severe wear with parafunction; posterior load-bearing	Superior strength; antagonist wear concerns	[35,36]
Tooth-supported full-arch FDPs	Generalized severe wear with adequate residual dentition	Tooth-borne biology; technically demanding; longevity depends on residual dentition	[35,36]
Implant-supported fixed prostheses	Edentulism or severely compromised dentition	Stable VDO; surgical morbidity; requires verified VDO before placement	[39]
All-on-4/full-arch implant-supported	Total or near-total edentulism	Long-documented survival; specialist-center evidence	[40]
Stabilization splint (post-rehabilitation)	Confirmed parafunction; protective adjunct	Reduces overload; compliance-dependent	[4,17]

Special clinical considerations

VDO Loss in the Older Patient

With advancing age, there is a notable decline in the adaptability of masticatory muscles, diminished salivary flow, and a rise in systemic comorbidities and polypharmacy. Additionally, changes in bone quality and increased sensitivity to alterations in occlusion and phonetics further complicate treatment [22,24]. These combined factors limit how much the VDO can be safely adjusted and raise the stakes when considering irreversible procedures.

In this population, a conservative and minimally invasive approach is especially vital [4]. Techniques such as direct composite restorations, removable prostheses designed with careful VDO considerations, and implant-retained overdentures often provide biomechanically sound and biologically favorable alternatives to full-arch fixed rehabilitations. Importantly, treatment planning must incorporate the patient’s overall health, cognitive status, social environment, and personal treatment goals. Establishing realistic expectations through collaboration with the patient and their medical team is crucial for successful outcomes.

VDO Loss and TMDs

The current evidence does not support a direct causal link between VDO loss and TMDs [27,28]. The Diagnostic Criteria for TMD (DC/TMD) framework views TMD as a complex, multifactorial biopsychosocial condition [27]. When managing patients presenting with both VDO loss and TMD symptoms, clinicians should prioritize reversible, evidence-based treatments such as cognitive behavioral therapy, physiotherapy, pharmacologic approaches, and stabilization splint therapy before contemplating any irreversible occlusal changes. Restoring VDO should not be considered a primary treatment for TMD unless there is a separate prosthodontic indication [28].

The Psychosocial Dimension

The psychosocial consequences of VDO loss often go underrecognized. Changes in facial esthetics can impact self-image, while functional impairments may hinder social eating and speech. Moreover, the psychological toll of progressive tooth wear and destruction can be substantial [41,42]. Patients with significant VDO reduction and disfiguring wear frequently report distress and lowered oral health-related quality of life, as measured by validated tools like the Oral Health Impact Profile (OHIP) [41,42]. Acknowledging these esthetic concerns and quality-of-life issues is essential for comprehensive treatment planning.

Research gaps and future directions

Absence of Standardized Diagnostic Criteria

One of the most critical gaps is the lack of universally accepted, clearly defined diagnostic criteria for VDO loss. Without such standards, it becomes challenging to compare findings across studies, making epidemiological prevalence estimates unreliable and complicating the design of robust clinical trials. Developing and validating standardized diagnostic protocols - ideally modeled after frameworks like the DC/TMD criteria used for TMDs [27] - should be a top research priority moving forward.

Longitudinal Outcome Data

An obvious deficiency exists in long-term outcome data following VDO rehabilitation, especially in prospective cohort studies with follow-ups extending beyond a decade. Key questions remain unanswered: How durable are restorative materials over time? Does the restored VDO remain stable in the long run? What is the incidence of prosthetic complications years after treatment? And crucially, how does long-term rehabilitation impact patients’ quality of life? Current evidence in these areas remains sparse and calls for more comprehensive, extended investigations.

Comparative Effectiveness Research

Research directly comparing the effectiveness of different diagnostic and treatment modalities for VDO management remains underdeveloped. Randomized controlled trials contrasting approaches - such as the Dahl technique versus indirect restorative methods, or staged full-arch rehabilitation against simultaneous procedures - could yield valuable, actionable insights. Such evidence would greatly enhance clinical decision-making by clarifying which strategies offer superior outcomes under various conditions.

Validation of Digital Technologies

While digital diagnostic tools for assessing VDO have shown promising precision [30,31], their clinical validation is still largely lacking. Without solid evidence confirming their accuracy and reliability in practice, these technologies should be viewed as complementary aids rather than replacements for established clinical assessment methods. Dedicated research efforts are needed to confirm their true clinical utility and guide their integration into standard care.

Patient-Reported Outcome Measures (PROMs)

Incorporating validated PROMs into VDO rehabilitation research is crucial to capture treatment success from the patient’s viewpoint, alongside traditional clinical metrics [41,42]. Future studies should routinely include PROM collection to ensure that the patient experience informs both research and clinical practice, ultimately fostering more patient-centered care.

Research on Adaptation

A fundamental question that remains largely unanswered is what governs an individual patient’s capacity to adapt to changes in VDO. Most studies exploring the neurophysiological, muscular, and proprioceptive factors underlying successful adaptation are sparse [2,6]. Addressing this gap could significantly improve personalized treatment planning and outcomes in prosthetic rehabilitation.

## Conclusions

Loss of VDO represents a clinically significant and diagnostically challenging condition that intersects multiple dental disciplines. VDO loss is a clinical syndrome with multifactorial causes rather than a single diagnostic entity; tooth wear, particularly erosive wear, remains the dominant contemporary driver. Loss of tooth substance does not necessarily mean VDO loss; dentoalveolar compensation can maintain VDO but may reduce freeway space. No single diagnostic parameter is reliable on its own; a holistic, integrative diagnostic approach is essential. The clinical decision-making should follow a reversible-to-irreversible pathway, employing provisional restorations or splints to validate the proposed VDO before definitive treatment. Treatment should aim for the minimum effective intervention; the smallest functional increase in VDO is generally preferable to maximal reconstruction. The evidence base remains uneven; clinical decisions require blending best available evidence with biological understanding and tailored patient judgment.

## Appendices

**Table 4:** Database search strategy.

Domain	Boolean Search String
1. Vertical dimension	("vertical dimension"[MeSH] OR "vertical dimension of occlusion" OR "occlusal vertical dimension" OR "freeway space" OR "interocclusal rest space")
2. Etiology	("tooth wear"[MeSH] OR "erosive tooth wear" OR "dental erosion" OR attrition OR abrasion OR abfraction OR bruxism[MeSH] OR "tooth loss" OR "combination syndrome" OR "posterior support")
3. Diagnosis	(BEWE OR "Basic Erosive Wear Examination" OR TWES OR "Tooth Wear Evaluation System" OR "closest speaking space" OR "interocclusal rest" OR cephalometric OR "intraoral scanning" OR "digital workflow" OR "diagnostic wax-up")
4. Treatment	("Dahl concept" OR "three-step technique" OR "occlusal veneer" OR "lithium disilicate" OR zirconia OR "full-mouth rehabilitation" OR "All-on-4" OR "occlusal splint" OR "composite resin" OR "adhesive rehabilitation")
Combined query	(Domain 1) AND ((Domain 2) OR (Domain 3) OR (Domain 4))
Filters	Humans; English language; 1975 to October 2025; Article types: Review, Systematic Review, Meta-Analysis, Randomized Controlled Trial, Clinical Trial, Comparative Study, Multicenter Study, Observational Study, Practice Guideline, Consensus Development Conference.
